# CPAP delivered via a helmet interface in lightly sedated patients with moderate to severe ARDS: predictors of success outside the ICU

**DOI:** 10.36416/1806-3756/e20240299

**Published:** 2024-11-16

**Authors:** Isabella de Melo Matos, Betina Santos Tomaz, Maria da Penha Uchoa Sales, Gabriela Carvalho Gomes, Antonio Brazil Viana, Miguel R. Gonçalves, Marcelo Alcantara Holanda, Eanes Delgado Barros Pereira

**Affiliations:** 1. Departamento de Medicina Interna, Universidade Federal do Ceará, Fortaleza (CE) Brasil.; 2. Hospital Universitário Walter Cantídio, Universidade Federal do Ceará, Fortaleza (CE) Brasil.; 3. Serviço de Pneumologia, Hospital de Messejana Dr. Carlos Alberto Studart Gomes, Fortaleza (CE) Brasil.; 4. Centro de Pesquisa Clínica, Universidade Federal do Ceará, Fortaleza (CE) Brasil.; 5. Unidade de Suporte de Ventilação Não Invasiva, Serviço de Pneumologia e de Emergência e Cuidado Intensivo, Centro Hospitalar de São João, Porto, Portugal.; 6. UnIC/RISE Cardiovascular R&D Unit, Faculdade de Medicina, Universidade do Porto, Porto, Portugal.

**Keywords:** Respiratory distress syndrome, COVID-19, Continuous positive airway pressure, helmets, Dexmedetomidine

## Abstract

**Objective:**

This study aimed to describe the outcomes and explore predictors of intubation and mortality in patients with ARDS due to COVID-19 treated with CPAP delivered via a helmet interface and light sedation.

**Methods:**

This was a retrospective cohort study involving patients with COVID-19-related ARDS who received CPAP using a helmet developed in Brazil (ELMO™), associated with a light sedation protocol in a pulmonology ward. Demographic, clinical, imaging, and laboratory data, as well as the duration and response to the ELMO-CPAP sessions, were analyzed.

**Results:**

The sample comprised 180 patients. The intubation avoidance rate was 72.8%. The lack of necessity for intubation was positively correlated with younger age, > 24-h continuous HELMET-CPAP use in the first session, < 75% pulmonary involvement on CT, and ROX index > 4.88 in the second hour. The overall in-hospital mortality rate was 18.9%, whereas those in the nonintubated and intubated groups were 3.0% and 61.2%, respectively. Advanced age increased the mortality risk by 2.8 times, escalating to 13 times post-intubation.

**Conclusions:**

ELMO-CPAP with light sedation in a pulmonology ward was successful in > 70% of patients with moderate to severe ARDS due to COVID-19. Younger age, pulmonary involvement, ROX index, and prolonged first Helmet-CPAP session duration were associated with no need for intubation. Older age and intubation are associated with mortality.

## INTRODUCTION

Respiratory involvement in COVID-19 has a clinical spectrum ranging from asymptomatic or oligosymptomatic patients to those with severe acute hypoxemic respiratory failure (AHRF) and ARDS.^1^ The persistence of the latter condition has remained the main risk factor for COVID-19-related endotracheal intubation (ETI) and mortality since the beginning of the pandemic.^2^ ^-^ ^4^ Noninvasive respiratory support strategies using helmet interfaces have become important for avoiding invasive measures.^5^ ^-^ ^7^ However, insistence on these strategies can delay ETI and worsen patient outcomes.^8^ ^,^ ^9^ Factors for predicting ETI and mortality continue to be a subject of debate.^10^ ^,^ ^11^

Helmet-CPAP has been used in ARDS patients with COVID-19 to provide prolonged and continuous treatments with high positive airway pressure (CPAP or PEEP) to reduce self-inflicted lung injuries.^12^ In association with noninvasive respiratory support, light sedation has been gaining prominence with the advantage of reducing anxiety, decreasing respiratory rate, and decreasing tidal volumes, factors that can aggravate lung injury if elevated, as well as improving patient compliance and tolerance to therapy.^13^ ^-^ ^15^ However, the association of CPAP with helmets and light sedation is limited to a few cases in the literature and little explored in ARDS outcomes.^13^ ^,^ ^16^

Helmet-CPAP is commonly implemented with an oxygen flow generator: it is simple to apply, does not require mechanical ventilators, and is usable outside the ICU.^13^ ^,^ ^17^ ^,^ ^18^ It is particularly useful in low-resource locations and during pandemics.^6^ ^,^ ^7^ ^,^ ^19^ ^,^ ^20^

During the second wave of the COVID-19 pandemic in Brazil, a helmet known as ELMO™ was used in thousands of patients. It was designed for the application of CPAP outside of overwhelmed ICUs during a pandemic emergency and does not require a mechanical ventilator.^21^ ^,^ ^22^ Respiratory wards were opened for non-intubated patients with AHRF with team training and structured protocols.

Patients with COVID-19 are especially anxious and frightened because of the disease. This factor potentially increases respiratory drive and respiratory rate and reduces patient tolerance to therapy.^1^ ^,^ ^23^ Therefore, we added pharmacological intravenous continuous light sedation with dexmedetomidine, a drug recommended for sedating patients during noninvasive respiratory support.^13^

We hypothesized that applying Helmet-CPAP with light sedation would augment patient tolerance, allowing prolonged periods of helmet application in patients with moderate to severe ARDS and COVID-19 treated outside the ICU, and that we would find predictors for ETI and mortality yet to be reported in the literature, such as the duration of Helmet-CPAP sessions. This study aimed to describe the outcomes of intubation and mortality and to explore their predictors in patients with ARDS secondary to COVID-19 treated with ELMO-CPAP (the specific helmet used in these patients) associated with light sedation with dexmedetomidine.

## METHODS

### Study design and population

This retrospective cohort study was performed at a specialized noninvasive respiratory support pulmonology ward for patients with AHRF and ARDS due to COVID-19 at the Hospital of Messejana Dr Carlos Alberto Studart Gomes, a tertiary referral hospital for respiratory diseases in the state of Ceará, Brazil. This study was performed in strict accordance with the Declaration of Helsinki and approved by the Brazilian National Research Ethics Committee (Protocol number. 4.902.781). The requirement for informed consent was waived owing to the retrospective nature of the study.

Eligible patients were adults > 18 years of age, of both sexes, diagnosed with COVID-19 confirmed by positive RT-PCR nasopharyngeal swab test,^24^ with hypoxemic respiratory failure submitted to noninvasive respiratory support therapy using ELMO-CPAP during the study period. ARDS was classified according to the new definition, which includes criteria such as assessment of the Pao_2_/FIo_2_ ratio when using CPAP > 5 cmH_2_O (201-300 = mild; 101-200 = moderate; and ≤ 100 = severe), bilateral opacities on chest CT, and a compatible underlying cause.^25^

The patients met the following criteria: 1. Being alert, oriented, and cooperative; 2. Oxygen therapy by nasal cannula with a flow > 4 L/min or by nonrebreathing reservoir mask > 8 L/min, maintaining SpO_2_ higher than 92%; 3. Arterial blood gas analysis with pH > 7.35 (no acidosis), Pao_2_ > 60 mmHg up to 30 min before starting therapy, and no hypercapnia (PaCo_2_ < 46 mmHg); and 4. Chest CT scan obtained within the preceding 24 h showing bilateral parenchymal opacities.^7^

Exclusion criteria were as follows: 1. first use of ELMO-CPAP after extubation; 2. Exacerbation of asthma or COPD; 3. Need for more than 0.5 mcg/kg/min of i.v. norepinephrine infusion; 4. Persistent signs of respiratory fatigue (use of accessory respiratory muscles and paradoxical breathing); 6. Uncontrolled claustrophobia; 7. Uncontained vomiting or nausea; and 8. Imminent risk of cardiorespiratory arrest.

**Figure. 1:**
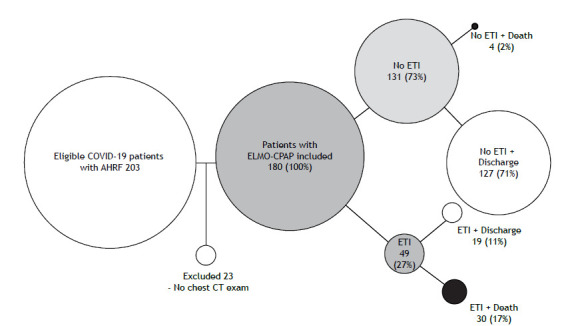
Flow chart of the study and outcomes. AHRF: acute hypoxemic respiratory failure; and ETI: endotracheal intubation.

The respiratory ward was monitored with a multiparameter monitor for vital signs (e.g. continuous pulse oximetry, frequency of blood pressure measurements). It was covered 24 h by doctors, nurses, and respiratory therapists, with a ratio of such professionals of 1:6 per patient, with daily shifts of fixed pulmonologists. The patients who underwent ELMO-CPAP support followed a standardized protocol (Figure 1) developed by the interdisciplinary team, all adequately trained and headed by a pulmonologist responsible for the continuous efforts to improve the team’s performance every day.

The ELMO-CPAP works by delivering a continuous flow of a mixture of oxygen and compressed medicinal gases directly into the inspiratory circuit; a PEEP valve in the exhalation port allows the application of a stable, adjustable CPAP of 8-15 cmH_2_O, which can be measured with a simple analog tracheal tube cuff pressure manometer.^21^

All patients started therapy with a total flow rate of 60 L/min (O_2_ and compressed air), with sufficient FIo_2_ to maintain an Spo_2_ in the range of 92-96%, and a CPAP of 10 cmH_2_O checked with the cuff pressure manometer.^22^

To reduce anxiety and improve patient compliance and comfort, patients were administered dexmedetomidine by continuous intravenous infusion (0.2-0.6 mcg/kg/min) and clonazepam oral drops (1 mg every 8 h) during ELMO-CPAP. Sedation was gradually adjusted to achieve a Richmond Agitation Sedation Scale score of 0 or −1.^5^ Some precautions were adopted as follows: the use of lubricating eye drops (before each CPAP session), nasal lavage with 0.9% sodium chloride saline, lactulose, and ear protectors. Spontaneous prone position was encouraged, adopted according to the patient’s tolerance, and maintained as long as possible. Figure S1 and S2 (supplementary material) shows details of the ELMO-CPAP application protocol and weaning from therapy, respectively.

### Data collection plan

Data were extracted from the electronic medical records using standardized forms, keeping the identity of each patient confidential. Data collection was performed by a single researcher who was adequately trained to use the data-collection instrument.

Baseline clinical data were collected on hospital admission. An arterial blood gas sample was collected before the start of therapy and during the first therapy session (with a time interval of 2-24 h of continuous ELMO-CPAP use). A qualitative description of chest CT performed within 24 h after admission was also analyzed by a board-certified radiologist who described the following items: degree of lung parenchymal involvement with findings suggestive of viral pneumonia (graduated as < 25%, 25-50%, > 50-75%, and > 75% of lung involvement). Subsequently, for statistical analysis, these categories were grouped as ≤ 50%, 51-75%, and > 75%. Complications such as pneumomediastinum or pneumothorax, if present, were recorded.

Data on clinical evolution of the disease were obtained, such as the beginning and type of symptoms before admission, time of therapy initiation after admission to the respiratory ward, total duration of ELMO-CPAP use in the first 48 h, and duration in days.

Patients who did not require ETI were considered successful, whereas those who needed it were considered failure.

The primary objective of this study was to assess the factors related to the success of ELMO-CPAP respiratory support. The secondary objective was to evaluate the factors associated with in-hospital mortality, including intubation.

### Statistical analysis

The normality test for quantitative variables was performed using the Shapiro-Wilk and Kolmogorov-Smirnov tests. For the descriptive analysis of quantitative variables, the mean and standard deviation or the median and interquartile range were calculated according to the sample distribution. Categorical variables were described as absolute and relative frequencies.

The Student’s t-test and Mann-Whitney U tests were used to compare continuous variables with normal and non-normal distributions, respectively. Association analysis between categorical variables was performed using the Pearson’s chi-square test.

Multivariate analysis and Poisson regression with robust estimator examined variables associated with ETI (primary outcome) and in-hospital death (secondary outcome). The variables considered relevant to the model were selected from those found significant in the univariate analyses (p < 0.05), according to clinical importance and biological plausibility, and others as potential confounders: age, C-reactive protein, chest CT involvement, Pao_2_/FIo_2_ ratio at baseline, and duration of first ELMO-CPAP section. The results are presented as relative risk (RR) and 95% CI. Statistical significance was set at p < 0.05. We used the IBM SPSS Statistics software, version 21.0 (IBM Corporation, Armonk, NY, USA).

## RESULTS

A total of 203 patients diagnosed with AHRF secondary to COVID-19 were admitted to the respiratory ward. All patients were eligible for ELMO-CPAP therapy. After applying the inclusion criteria, 180 patients were included in the analysis (Figure 1). Of these, 116 (81%) were male, and the median age of the sample was 55 years. Table 1 shows the patient characteristics, treatments applied, and hospital outcomes of the nonintubated (success) and intubated (failure) groups. A total of 131 (72.8%) patients did not need ETI after ELMO-CPAP, while 49 (27.2%) failed and received invasive mechanical ventilation.

**Table 1:** Patient characteristics, treatments applied, and hospital outcomes comparing the nonintubated (success) with the intubated (failure) groups.^a^

Variables	Whole sample	Group		p
		Nonintubated	Intubated	
	N = 180	n = 131	n = 49	
Demographic data				
Age, years	55 (45-63)	54 (41-62)	57.5 (50.0-67.0)	0.01
Male	116 (81.0)	87 (66.4)	29 (59.2)	0.38
SOFA score	2 (2-2)	2 (2-2)	2 (2-3)	0.06
Comorbidities				
Hypertension	77 (42.8)	53 (40.4)	24 (48.9)	0.34
Diabetes mellitus	51 (28.3)	34 (25.9)	17 (34.6)	0.24
Obesity	46 (25.5)	36 (27.4)	10 (20.4)	0.33
Heart failure	22 (12.2)	17 (12.9)	5 (10.2)	0.61
Cerebrovascular accident	3 (1.7)	1 (0.7)	2 (4.0)	0.12
Atrial fibrillation	2 (1.1)	2 (1.5)	0 (0.0)	0.38
COPD	5 (2.8)	2 (1.5)	3 (6.1)	0.95
Asthma	6 (3.3)	6 (4.5)	0 (0.0)	0.12
Anxiety	4 (2.2)	3 (2.2)	1 (2.0)	0.92
Other	17 (9.4)	12 (9.1)	5 (10.2)	0.83
None	53 (29.4)	43 (32.8)	10 (20.4)	0.10
Radiological and laboratory data				
Pulmonary involvement on chest CT				
≤ 50%	39 (21.7)	33 (25.2)	6 (12.2)	0.03
51-75%	101 (56.1)	77 (58.0)	24 (49.0)	
> 75%	40 (22.2)	21 (16.0)	19 (38.8)	
Hemoglobin = 13-18 g/dL	13.5 (12.6-14.5)	13.6 (12.6-14.5)	12.5 (11.0-13.1)	0.12
Haematocrit = 40-54%	40.4 (37.7-43.0)	40.8 (37.9-43.3)	39.3 (36.8-42.8)	0.39
Leucocytes = 4,000-10,000/mm3	9050 (7000-12350)	9700 (7600-13100)	7700 (5850-9750)	0.10
Lymphocytes = 1,500-4,500/mm3	864 (630-1170)	911 (634-1283)	791 (600-975)	0.02
D-dimer < 0.500 µg/mL)	810 (530-1160)	795 (508-1127)	870 (643-1450)	0.36
LDH = 140-271UI/L	381 (303-512)	365 (285-470)	407 (261-589)	0.11
C-reactive protein, mg/dL	9.6 (6.52-14.6)	9.0 (6.2-14.5)	11.9 (9.1-15.2)	0.01
Urea, mg/dL	32 (25-42)	32 (24-41)	31 (26-43)	0.84
Creatinine, mg/dL	0.8 (0.66-0.96)	0.7 (0.6-0.9)	0.8 (0.6-1.0)	0.41
Arterial blood gas analysis				
pH	7.45 (7.43-7.47)	7.45 (7.43-7.47)	7.45 (7.42-7.48)	0.55
PaCO_2_, mmHg	35.8 (32.9-38.2)	36.8 (33.4-39.1)	33.7 (31-38.0)	0.05
PaO_2_, mmHg	76 (67.5-89.0)	76 (68.9-90.5)	76.2 (62.1-85.3)	0.18
SaO_2_, %	95.2 (93.5-96.6)	96 (94-98)	95 (93.0-96.7)	0.008
Lactate, mmol/L	1.68 (1.28-2.39)	1.7 (1.2-2.4)	1.5 (0.9-2.3)	0.54
PaO_2_/FIO_2_	138 (116.5-163)	142.5 (121.0-163.0)	117.5 (100.0-141.0)	0.001
Concomitant medications				
Dexamethasone	180 (100)	131 (100)	49 (100)	NA
Albendazole or ivermectin	180 (100)	131 (100)	49 (100)	NA
Deep vein thrombosis prophylaxis	167 (92.7)	107 (81.6)	25 (51.0)	0.01
Anticoagulation	13 (7.2)	24 (18.3)	24 (48.9)	0.01
Antibiotics	173 (96.1)	124 (94.7)	49 (100)	0.1
Days of symptoms preceding ELMO-CPAP use	10 (8-12)	10 (8-12)	10 (7.5-11.5)	0.86
Duration of 1st ELMO-CPAP session, h	39 (24-48)	44 (32.2-48.0)	24 (17-42)	< 0.001
Total duration of ELMO-CPAP, days	4 (2-5)	4 (3-5)	3 (2-4)	0.104
Outcomes				
Length of hospital stay, days	13 (9-23)	11 (9-15)	25 (20-32.7)	< 0.01
In-hospital mortality	34 (18.9)	4 (3.0)	30 (61.2)	0.001
Complications				
Pneumothorax	2 (1.1)	0 (0.0)	2 (4.1)	0.001
Pneumomediastinum	9 (5.0)	5 (3.8)	4 (8.2)	0.23

a Data expressed as n (%) or median (IQR).

Older age [57.5 years (50-67) vs. 54 years (41.2-62.0); p = 0.01], higher levels of C-reactive protein [11.9 mg/L (9.1-15.2) vs. 9 mg/L (6.2-14.5); p = 0.01], lower Pao_2_/FIo_2_ ratio [117.5 (100.0-141.0) vs. 142.5 (121.0-163.0); p = 0.001], more than 75% of pulmonary involvement on chest CT images [19 (38.8%) vs. 21 (16%); p = 0.03], and fewer hours during the first ELMO-CPAP session [24 h (17-42) vs 44 h (32.2-48.0); p < 0.001] were associated with ETI in univariate analysis (Table 1). All patients used light sedatives, as described in the study protocol.

The length of hospital stay for intubated patients was more than double of that for nonintubated patients [25 days (20.0-32.7) vs. 11 (9.0-15.0); p < 0.01]. Overall, the in-hospital mortality was 18.9%, and there was a statistically significant difference between the intubated and nonintubated groups [30 (61.2%) vs. 4 (3%); p = 0.001; Table 1].

Considering the secondary outcome, when comparing death versus hospital discharge, mortality was higher in older patients who presented with higher levels of C-reactive protein, LDH, and D-dimer; those who were hyperventilated; those with lower levels of Pao_2_/FIo_2_ ratio; those who presented more than 75% of pulmonary involvement on chest CT images; and those who spent fewer continuous hours during the first ELMO-CPAP session (28 h vs. 42 h; p = 0.048), as detailed in Table S1.

Table 2 shows the response to ELMO-CPAP in arterial blood gases and Spo_2_ collected at 2-24 h, variation to baseline, and respiratory rate-oxygenation (ROX) index at 2 h during the first ELMO-CPAP application in nonintubated and intubated patients. An improvement in oxygenation was observed in the nonintubated group, which was reflected in the increase in Pao_2_, Spo_2,_ and Pao_2_/FIo_2_ during the use of ELMO-CPAP. In addition, there was an early response in the ROX index after 2 h, with a median of 5.56 vs 4.84 (p = 0.003) in nonintubated and intubated patients, respectively. Most patients had moderate to severe ARDS. The latter was more frequent in the intubation group and was associated with failure (Table 2).

**Table 2:** Arterial blood gas analysis and SpO_2_ collected at 2-24 h after admission, variation in relation to baseline, and respiratory rate-oxygenation index at 2 h during the first ELMO-CPAP application.^a^

Variable	Whole sample	Group		p
		Nonintubated	Intubated	
	N = 180	n = 131	n = 49	
Arterial blood gas (ABG)				
pH	7.44 (7.41-7.47)	7.44 (7.41-7.47)	7.44 (7.42-7.47)	0.71
PaCO_2_, mmHg	37.3 (33.3-40.9)	37.7 (34.5-41.0)	36.4 (32.8-39.9)	0.20
PaO_2_, mmHg	90.1 (75.6-115-7)	94 (77-119)	83 (69.9-107.3)	0.03
PaO_2_/FIO_2_	160 (125-205)	176 (135-221)	127 (106-159)	0.01
Lactate, mg/dL	1.79 (1.38-2.34)	1.8 (1.35-2.34)	1.78 (1.57-2.38)	0.41
SpO_2_, %	97 (95-98)	97 (95-98)	95.9 (93.7-97.9)	0.03
ARDS classification				
Mild (PaO_2_/FIO_2_ = 300-201)	30 (16.7)	25 (19.1)	5 (10.2)	
Moderate (PaO_2_/FIO_2 =_ 200-101)	108 (60.0)	78 (59.5)	30 (61.2)	
Severe (PaO_2_/FIO_2_ ≤ 100)	19 (10.6)	8 (6.1)^a^	11 (22.4)^b^	0.005
Variation in ABG in relation to baseline				
ΔpH	−0.01 (−0.04 to 0.01)	to 0.01 (−0.04 to 0.01)	to 0.01 (−0.05 to 0.02)	0.14
ΔPaCO_2_	1.5 (−0.97 to 4.8)	1.5 (−1.0 to 4.8)	1.9 (−1.2 to 4.45)	0.37
ΔPaO_2_	13.2 (−2.71 to 37)	14.3 (−2.0 to 40.5)	16.9 (−8.5 to 38.5)	0.96
ΔPaO_2_/FIO_2_	20 (−10.7 to 61.5)	25 (−11 to 64)	16 (−16 to 60)	0.61
Δlactate	−0.34 (−0.81 to 0.22)	0.09 (−0.43 to 0.81)	−0.19 (−1.0 to 0.5)	0.86
ΔSpO_2_	1.2 (−0.4 to 3.27)	1.3 (−0.3 to 3.7)	1.3 (−1.95 to 3.3)	0.79
ROX^b^ index at 2 h	5.39 (4.3-6.1)	5.56 (4.63-6.15)	4.84 (4.08-5.75)	0.003

ROX: respiratory rate-oxygenation. ^a^Data expressed as n (%) or median (IQR). ^b^The ROX index is defined as the ratio SpO_2_/FIO_2_ to the respiratory rate. In case of missing data, statistics were performed on available data. Differences in frequencies were tested with the chi-square test. Differences in continuous variables were tested with the Mann-Whitney test. ^a^ Chi-square test and post hoc analysis for pairwise comparisons for success group. ^b^ Chi-square test and post hoc analysis for pairwise comparisons for failure group.

Multivariate analysis with Poisson regression was used to examine the factors associated with ETI and mortality. The relevant variables for the model were age, C-reactive protein level, involvement on chest CT, Pao_2_/FIo_2_ ratio at the beginning of the study, and duration of the first ELMO section.

**Figure 2:**
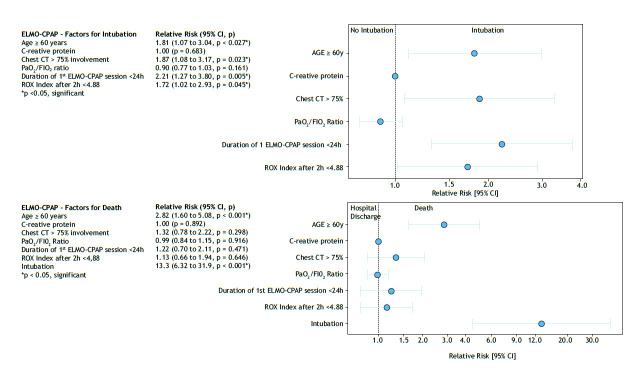
Multivariate logistic regression analysis with independent variables of factors for intubation and death.

For intubation, multivariate analysis showed that patients over 60 years of age, with more than 75% pulmonary involvement on chest CT images, an ROX index < 4.88, and less than 24 h duration of the first ELMO-CPAP session were associated with a higher risk for intubation (Figure 2).

Regarding mortality, advanced age was associated with an increase of 2.8 times in the risk of mortality, and this risk escalated to a 13-time increase in individuals who underwent intubation. The other variables were not associated with mortality (Figure 2).

## DISCUSSION

This retrospective cohort study included 180 patients with AHRF and ARDS secondary to COVID-19 treated with CPAP without mechanical ventilation using the first Brazilian helmet interface (ELMO), light sedation, and outside the ICU. In our sample, 131 (72.8%) of the patients did not need ETI. To our knowledge, this is the highest success rate reported in the literature. The overall in-hospital mortality rate was 18.9%, and almost all deaths occurred in the intubation group.

The independent predictive factors associated with ETI were old age, > 75% pulmonary involvement on chest CT images, ROX index < 4.88 after 2 h, and duration of first ELMO-CPAP session less than 24 h. Advanced age was associated with an increase of up to 2.8 times in the risk of death, and this risk escalated by more than 10 times in individuals who underwent intubation.

The application of noninvasive respiratory support outside the ICU in the patient profile of this study is feasible when performed by an experienced, trained team and is associated with favorable outcomes, such as lower rates of intubation and mortality.^10^ ^,^ ^19^ These settings have become known as specialized wards, with institutional protocols and well-defined criteria for initiating therapy, guided by pulmonologists and a multidisciplinary team. These factors may be associated with a learning curve for the team in managing ELMO-CPAP, thereby influencing the high success rate. Compared with other studies that used CPAP with a helmet interface, our success rate is currently the highest reported in the literature. Previously, other researchers reported success rates of 63%,^26^ 69%,^27^ and 56%.^28^

The high nonintubation rate in patients using ELMO-CPAP with light sedation obtained in our investigation can be explained by the improvement in oxygenation, probably due to alveolar recruitment, improvement in the ventilation-perfusion ratio, and dyspnea relief.^12^ This result can be potentiated with helmet-type interfaces, such as ours, by providing a greater tolerance for high CPAP levels for prolonged periods. To the best of our knowledge, the present study is the first to routinely use light sedation with Helmet-CPAP in all patients, which may have contributed to their success. Light sedation may favorably modulate the respiratory drive, a factor that, in theory, may contribute to reducing the propensity to self-inflicted lung injury.^12^ Dexmedetomidine is the preferred agent for sedating patients receiving Helmet-CPAP in the ICU when necessary and promotes lower rates of delirium.^13^ ^,^ ^29^ In previous similar studies, the overall in-hospital mortality rates were 34.3%^30^ and 36.3%^26^ compared with 18.9% in the present investigation. The relatively lower mortality rate in our study may be related to the lower ETI rate.

Two factors related to the patient and disease extension were independently associated with intubation: older age and extensive lung parenchymal involvement on chest CT, respectively. In fact, for every one-year increase in lifetime, the risk of ETI and mortality increased. This is consistent with the study by De Vita et al.,^31^ in which age and markers of disease severity, leukocytes, LDH, and Pao_2_/FIo_2_ were predictive factors of CPAP failure. In our study, lung parenchymal involvement on chest CT images < 75% at hospital admission was associated with an increased chance of no intubation. Patients with COVID-19 and greater extension of lung parenchyma infiltrates on chest CT have a reduced surface area for respiratory gas exchange with worsening during the course of the disease, which can lead to ETI.^32^ Considerable lung involvement can also predispose patients to complications such as pneumothorax and the need to be admitted to the ICU.^33^

We found a direct relationship between prolonged duration of the first ELMO-CPAP session and avoidance of ETI: a session for more than 24 h reduced the risk of intubation. Of the four retrospective cohort studies published to date,^11^ ^,^ ^30^ ^,^ ^31^ ^,^ ^34^ none associated the duration of continuous CPAP use in the first session with a decrease in intubation rate in patients with COVID-19 and AHRF, a new finding of the present investigation.

Our study has several limitations. It had a retrospective, single-center design, which limits the generalizability of the results. Arterial blood gas measurements were obtained within a broad timeframe of 2-24 h from therapy initiation, a software-based quantitative analysis of lung involvement on chest CT images was not used, and the measurement of the ROX index was performed at a single point in time. Therefore, it was not possible to include a control group in this study. However, the protocol and respiratory support were standardized for a single disease, limiting the variability of treatment and the number of confounding variables.

The clinical implications of this study are as follows. This study provides new predictors of intubation and mortality using Helmet-CPAP in severely ill patients with ARDS due to SARS-CoV-2. Furthermore, CPAP therapy, which is much simpler than noninvasive ventilation with pressure-support ventilation, can be applied in scenarios of public health emergencies or catastrophes with favorable outcomes, mainly in low-income communities or countries. Moreover, predictors related to the patient, disease extension, tolerance, and response to Helmet-CPAP use can help identify patients who need more attention and early recognition of failure, avoiding delayed intubations and possible increases in mortality.^35^ Future clinical trials are warranted to study the applications of Helmet-CPAP designed to implement the earliest and most prolonged duration possible in patients with AHRF and ARDS due to viral pneumonia or other causes combined with light sedation with dexmedetomidine or other sedatives, whenever needed, in comparison with alternatives such as high-flow nasal cannula or standard noninvasive ventilation.^36^

In conclusion, the use of a new helmet, ELMO, with CPAP and light sedation applied outside the ICU, resulted in more than 70% of COVID-19 patients with AHRF and ARDS not requiring ETI. Intubation and mortality rates were higher for elderly patients with more than 75% pulmonary involvement on chest CT images and less than 24 h duration for the first ELMO-CPAP session. The design of future investigations with Helmet-CPAP use in ARDS and AHRF should consider the present results.
